# The Phenotypic Spectrum of DYT24 Due to ANO3 Mutations

**DOI:** 10.1002/mds.25802

**Published:** 2014-01-17

**Authors:** Maria Stamelou, Gavin Charlesworth, Carla Cordivari, Susanne A Schneider, Georg Kägi, Una-Marie Sheerin, Ignacio Rubio-Agusti, Amit Batla, Henry Houlden, Nicholas W Wood, Kailash P Bhatia

**Affiliations:** 1 Sobell Department of Motor Neuroscience and Movement Disorders, University College London (UCL) Institute of NeurologyLondon, United Kingdom; 2 Neurology Clinic, Attiko Hospital, University of AthensGreece; 3 Department of Molecular Neuroscience, UCL Institute of NeurologyLondon, United Kingdom; 4 Department of Clinical Neurophysiology, National Hospital for Neurology and NeurosurgeryLondon, United Kingdom; 5 Department of Neurology, University of KielKiel, Germany; 6 Department of NeurologyKantonsspital St. Gallen, St. Gallen, Switzerland

**Keywords:** cervical dystonia, cranial dystonia, laryngeal dystonia, dystonic tremor, *ANO3*, DYT24

## Abstract

Genes causing primary dystonia are rare. Recently, pathogenic mutations in the anoctamin 3 gene (*ANO3*) have been identified to cause autosomal dominant craniocervical dystonia and have been assigned to the dystonia locus dystonia-24 (DYT24). Here, we expand on the phenotypic spectrum of DYT24 and provide demonstrative videos. Moreover, tremor recordings were performed, and back-averaged electroencephalography, sensory evoked potentials, and C-reflex studies were carried out in two individuals who carried two different mutations in *ANO3*. Ten patients from three families are described. The age at onset ranged from early childhood to the forties. Cervical dystonia was the most common site of onset followed by laryngeal dystonia. The characteristic feature in all affected individuals was the presence of tremor, which contrasts DYT24 from the typical DYT6 phenotype. Tremor was the sole initial manifestation in some individuals with *ANO3* mutations, leading to misdiagnosis as essential tremor. Electrophysiology in two patients with two different mutations showed co-contraction of antagonist muscles, confirming dystonia, and a 6-Hz arm tremor at rest, which increased in amplitude during action. In one of the studied patients, clinically superimposed myoclonus was observed. The duration of the myoclonus was in the range of 250 msec at about 3 Hz, which is more consistent with subcortical myoclonus. In summary, *ANO3* causes a varied phenotype of young-onset or adult-onset craniocervical dystonia with tremor and/or myoclonic jerks. Patients with familial cervical dystonia who also have myoclonus-dystonia as well as patients with prominent tremor and mild dystonia should be tested for *ANO3* mutations. © 2014 The Authors. Movement Disorders published by International Parkinson and Movement Disorder Society

Recently, using whole-exome sequencing and linkage analysis, pathogenic mutations of the *ANO3* gene (encoding for anoctamin 3) were identified as a new cause of primary familial craniocervical dystonia in an autosomal dominant kindred.^1^ *ANO3* (Mendelian Inheritance in Man number, 610110; National Center for Biotechnology GeneBank Reference Sequence number, NM_031418) is located on chromosome 11, consists of 27 exons, and is highly expressed in the striatum.^1^ By homology with other members of this gene family, anoctamin 3 is a probable Ca^2+^-gated chloride channel, and functional studies using Ca^2+^ imaging in mutation-bearing and control fibroblasts demonstrated clear abnormalities in endoplasmic reticulum-dependent Ca^2+^ signalling.^1^

Here, we describe and illustrate with videos the phenotypic spectrum of DYT24 due to *ANO3* mutations in the identified familial cases. We discuss the clinical implications and compare these cases with the phenotypes of dystonia-1 (DYT1); DYT6; guanine nucleotide binding protein (G protein) alpha activating polypeptide, olfactory type (GNAL); and DYT11 dystonia.

## Patients and Methods

### Standard Protocol Approvals, Registrations, and Patient Consents

The study was approved by the University College London Hospitals/University College London ethics committee (University College London Hospitals project ID number 06/N076), and informed consent was given by all family members.

### Patients

**FIG. 1:**
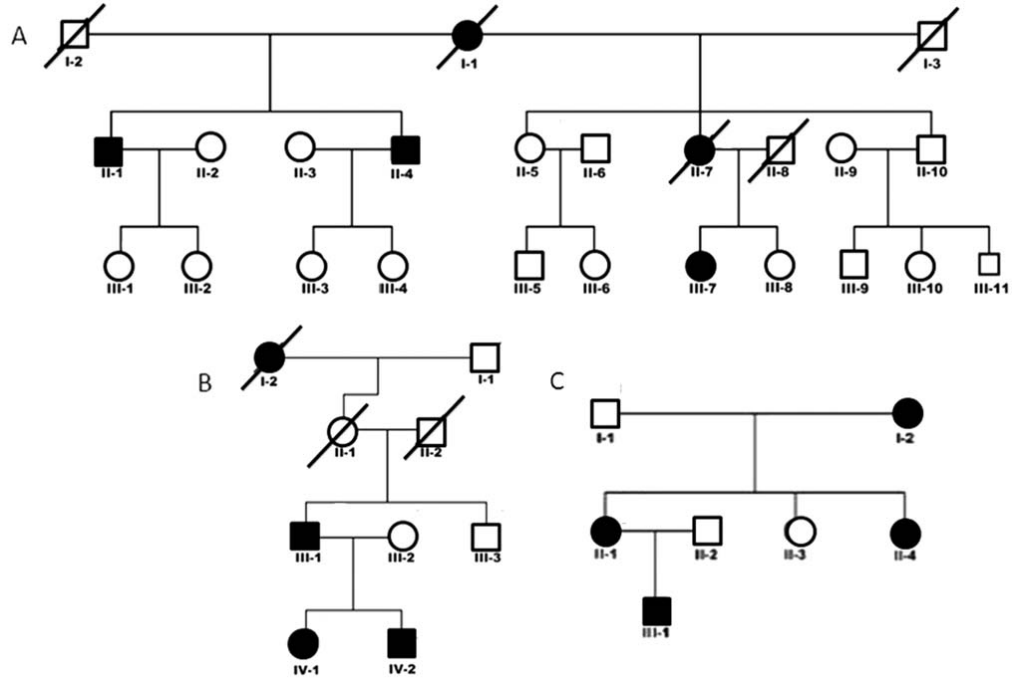
The family pedigrees are shown for (A) Family 1, (B) Family 2, and (C) Family 3.

In total, 384 patients with sporadic (n = 247) and familial (n = 137) craniocervical dystonia were screened for mutations in exon 15 of the *ANO3* gene.^1^ Of these, 112 patients (approximately 29%) had tremulous dystonia, and approximately 71% had nontremulous dystonia. One hundred seventy-five of 384 patients (45.5%) had adult-onset dystonia (at age >25 years), and 54.5% had young-onset dystonia. Forty of 384 patients had generalized dystonia. One hundred ninety of 384 patients were screened for mutations in all 27 exons of *ANO3*. Three families (10 individuals) were identified that harbored novel, putatively pathogenic mutations in *ANO3*. The description of these patients is provided below (see Results) and the family pedigrees are given in Fig. 1A,1B and 1C.

### Electrophysiology

Apart from dystonia, all patients also had tremor. Therefore, we obtained electromyography (EMG) recordings to characterize the tremor in two patients who had different mutations in *ANO3*. Moreover, superimposed myoclonus was noted in two patients. In one of these, we performed specialized electrophysiology to characterize the nature of the myoclonus, as outlined below.

### EMG and Back-Averaged Electroencephalography

Surface EMG was recorded from the involved muscles, which were chosen based on clinical observation of the most affected body parts using bipolar silver/silver chloride electrodes placed 2 cm apart over the belly of the muscles. Electroencephalography (EEG) was recorded from electrodes positioned on the frontocentral areas (International 10-20 system) with midline frontal (Fz) as the reference. Recordings were obtained at rest, with arms outstretched, and during action. One of the muscles most regularly involved in each jerk was selected to trigger the collection of EEG data for back-averaging. Only muscles with a well-defined burst of activity in each jerk were chosen. The video EEG-EMG recording was performed for about 60 minutes. Bilateral frontocentral regions (F4-C4, C4-Cz, F3-C3, C3-Cz) and cranial and limb muscles (orbicularis oculi, left sternocleidomastoid [SCM], right levator scapulae [LS], deltoid, biceps, extensor carpi radialis [ECR], flexor carpi radialis [FCR], first dorsal in-terosseus [1DIO], and rectus abdominis) were simultaneously recorded.

### Median Nerve Somatosensory Evoked Potential and C Reflex

The median nerve was stimulated at the left wrist with square wave pulses of 200-msec pulse width at just above the motor threshold at 2 Hz. Silver-silver chloride recording electrodes were placed on C30 (2 cm behind C3), C40 (2 cm behind C4) contralateral to the side of stimulation, and Fz (midline frontal). The filter band pass was 0.5 Hz to 1 kHz, and 1,000 sweeps were averaged. The amplitudes of the N20, P25, and N35 peaks were determined from the C30-Fz or C40-Fz recordings. The long loop reflex (C reflex) was simultaneously recorded on the thenar muscles from the median nerve stimulation at the wrist.

## Results

Routine magnetic resonance imaging studies and basic investigations for secondary/heredodegenerative dystonias were carried out in at least one affected member of each family and were normal. Genetic tests for DYT1, DYT6, and DYT11 (including large deletions) using standard published methods^2^ were performed and were negative. None of the patients were previously exposed to neuroleptic drugs.

### Family 1

Family 1 is the index family in which the gene was identified.^1^,^3^ Clinical details of this family have been published elsewhere (Fig. 1A; Video Segment 1). Age at onset ranged from 19 to 40 years (Table 1). The affected members of this family have a missense mutation in exon 15 (c.1480A>T; p.R494W) of the *ANO3* gene that is segregated in the affected family members and absent in unaffected family members.^1^

**TABLE 1:** The clinical features and evolution of the 10 affected members from 3 families

Individual (sex/age, y)	Age of Onset. y	Follow-Up, y	Site of Onset	Cervical Dystonia	Cranial Dystonia	Upper Limb Dystonia	Tremor	Myoclonus
Family 1								
I-1 (F/deceased)	27	—	Cervical	Torticollis	Laryngeal dysphonia, adductor dysphonia	—	Head, mild bilateral postural arms	
II-1 (M/age 67)	40	27	Cervical	Torticollis to the right	Blepharospasm, adductor dysphonia	—	Head, mild bilateral postural asymmetric arms	
II-4 (M/69)	30	39	Laryngeal	Torticollis	Blepharospasm, adductor dysphonia, oromandibular	Mild	Head; mild on the left arm	
II-7 (W/deceased)	30	27	Cervical	Torticollis	Adductor dysphonia	—	Head, bilateral postural asymmetric arms	
III-7 (W/36)	19	17	Cervical	Torticollis	Adductor dysphonia	Mild	Head	
Family 2								
III-1 (M/65)	3-4	61	Cervical	Torticollis	Laryngeal, oromandibular, blepharospasm	—	Head, mild bilateral postural asymmetric arms	
IV-1 (F/39)	4-5	30	Laryngeal	Torticollis	Laryngeal, oromandibular, blepharospasm	Mild fingers bilateral	Finger tremor and asymmetric arm tremor	Torticollis and retrocollic myoclonic jerks
IV-2 (M/37)	30	7	Right arm	-	-	Mild fingers only on the right	Mild postural tremor, more on the right arm	
Family 3								
II-1 (F/31)	6	25	Cervical	Torticollis	Laryngeal	Mild	Mild, postural and action, asymmetric arms; head	
III-1 (M/9)	6	3	Left arm	No	No	-	Moderate, mainly on action, asymmetric arms	Proximal and distal myoclonic jerks (arms)

F, female; M, male.

Patient II-4 (Table 1, Video Segment 1A), with intermittent blepharospasm and torticollis to the left, had electrophysiology of neck and limb muscles that showed continuous muscle over-activity in the levator scapulae, ECR, and FCR. There was a mild limb co-contraction tremor of the neck and the arms with the hand supported in semisupination, against gravity and during action, at about 6 Hz. There were no interruptions of muscle activity when holding both arms against gravity. Auditory stimulation (tapping stimulation of the mantle area) did not produce any abnormal startle response. EEG back-averaging was not possible because of the dystonic pattern underlying the muscle bursts.

### Family 2

Patient III-1 (age 65 years) (Fig. 1B) first noticed an intermittent head tremor at the age of 4 years and recalls being teased for this at school. Gradually, the head tremor became constant and was exaggerated by stress, while alcohol did not produce any improvement. In his teens, his voice became tremulous; and, by adulthood, his writing had become shaky. On examination at age 53 years, he had a side-to-side head tremor and oromandibular movements. He had voice tremor and a mild, fine postural tremor more on the right arm. The head tremor responded well to botulinum toxin injections. Examination at age 65 years showed no further progression of his symptoms.

**FIG. 2:**
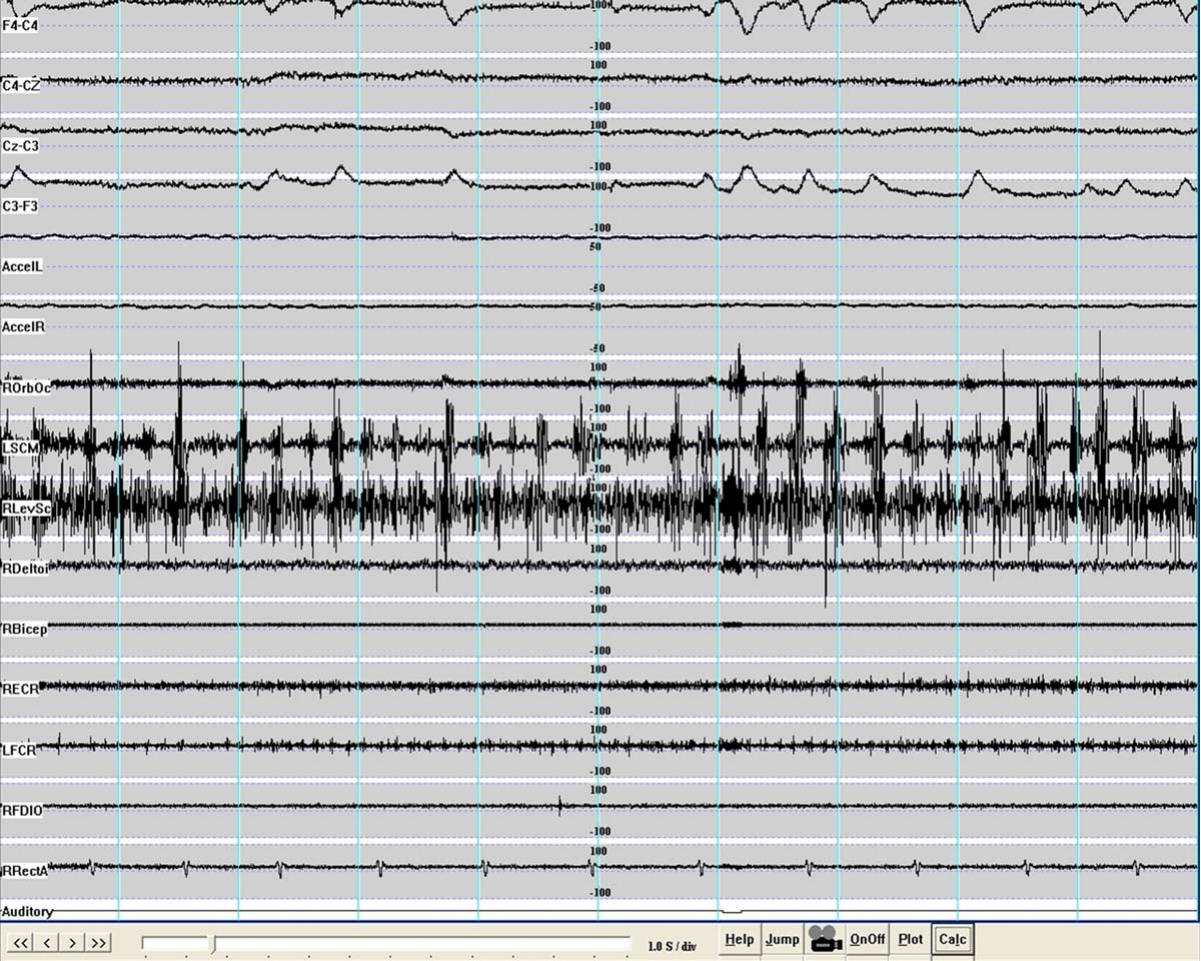
The dystonic activity of patient IV-1 (Family 2) is illustrated. There is intermittent activity in the orbicularis occuli muscles clinically presenting with blepharospasm. There is tremoulous torticollis to the right with continuous activity in the left sternocleidomastoid (LSCM) and the right levator scapulae (RLevSc) muscle. There is dystonic tremor at 6 Hz, superimposed myoclonic jerks at 3 to 4 Hz, and duration no shorter than 250 msec. There was underlying, continuous muscle over-activity in the extensor carpi radialis (ECR) and the flexor carpi radialis (FCR) [Color figure can be viewed in the online issue, which is available at http://wileyonlinelibrary.com.].

His daughter, patient IV-1 (age 39 years), developed laryngeal dysphonia at the age of 5 years and, shortly thereafter, difficulties with her jaw. At age 14 years, she developed tremor of her head and arms. Alcohol had no influence on the tremor. On examination at age 39 years, she had a torticollis to the left and head tremor. She had occasional finger and arm tremor on arms outstretched and occasional superimposed myoclonic jerks. She had laryngeal dysphonia and mild oromandibular dystonia (Video Segment 2A). Electrophysiology on this patient showed intermittent blepharospasm (Fig. 2) and torticollis to the right. There was underlying, continuous muscle over-activity in the levator scapulae, ECR, and FCR. There was tremor of the neck, trunk, and limbs. There was a mild limb co-contraction tremor with the hand supported in semisupination, against gravity and during action, at about 6 Hz. The myoclonic jerks of the neck were mostly torticollic rather than retrocollic. The duration of myoclonus was about 250 msec with a variable frequency of 3 to 4 Hz at rest. There were no interruptions of muscle activity when holding both arms against gravity. Auditory stimulation (tapping stimulation of the mantle area) did not produce any abnormal startle response. EEG back-averaging was not possible because of the dystonic pattern underlying the muscle bursts, which nonetheless were of long duration (250 msec). An investigation of somatosensory evoked potentials did not indicate any giant evoked potentials (P25-N35, 4 μV; N20-P25, 6.6. μV).

Patient IV-2 is 37-year old man who developed a bilateral (more on the right) postural hand tremor at the age of 30 years. On examination at age 37, he had a mild voice tremor, fine postural arm tremor more on the right, and a mild dystonic posturing of the fingers on the right (Video Segment 2B).

The affected members of this family carry a heterozygous missense mutation (c.1470G>C; p.W490C) in the *ANO3* gene.^1^ Interestingly, the grandmother of patient III-1, but not the mother, reportedly had head tremor. Although both individuals are now deceased and could not be examined, this suggests the possibility of reduced penetrance.

### Family 3

Patient II-1 (Fig. 1C) is a 31-year old woman who developed tremor of her head and intermittent tremor of her hands, mostly when under stress, at age 6 years. At age 19 years, she noticed problems with her voice. At age 20 years, she was diagnosed with essential tremor (ET). She was started on propranolol with no improvement and, later, received clonazepam, which had no effect. On examination (at age 31 years), she had a no-no head tremor, which was exaggerated with action and under stress. She had adductor laryngeal dysphonia (Video Segment 3A). She had a mild postural and action tremor more on the right arm, which would appear only in particular situations. The remainder of the clinical examination was unremarkable. Her 9-year old son (patient III-1) noticed a tremor of his hands more on the left, at age 6 years. On examination at age 9 years, he had proximal and distal myoclonus in his outstretched arms and tremor, more on the left and mainly when performing motor tasks (Video Segment 3B). The rest of the examination was normal.

Interestingly, the 65-year old grandmother (patient I-2) developed head and arm tremor at age 7 years, with no affection of her voice, and was diagnosed with ET. The younger sister (patient II-4; age 30 years) is said to have a voice tremor. However, both the grandmother and the younger sister reside outside the United Kingdom and were not available for examination. Of interest, patient II-1, who underwent surgery for an Arnold-Chiari malformation and has a son with autism (who was not affected by dystonia and was not tested for *ANO3* mutations), had a previous termination of pregnancy due to anencephaly of the fetus and has a nephew from her affected sister with agenesis of the corpus callosum (who was not tested for *ANO3* mutations). It is unknown whether these developmental malformations could represent associated features of *ANO3* dysfunction or are purely coincidental.

The affected members of this family carry a mutation in exon 21 of *ANO3* (c.2053A>G, p.S685G).

## Discussion

Here, we expand on the phenotypic manifestations of DYT24 caused by mutations in the newly identified dystonia gene *ANO3*. These comprise a spectrum, from a variable combination of cervical dystonia with tremor (Family 1 and Family 2 [patients III-1, IV-1], Family 3 [patient II-1]), cranial dystonia (blepharospasm, oromandibular dystonia) and laryngeal dystonia (Family 1, Family 2, and Family 3 [patient II-1]) to initially isolated and later predominant arm tremor with only minimal dystonic posturing of the fingers (Family 2 [patient IV-2], Family 3 [patient III-1]) and myoclonus of subcortical origin (Family 2 [patient IV-1], Family 3[patient III-1]). The range of age at onset varies from early childhood to the forties, implying that this gene may play a role in both young-onset and adult-onset dystonia. The cervical region was the most common site of onset, followed by laryngeal dystonia, and then the arms; whereas the legs were never affected. In most patients, dystonia progressed slowly over many years and spread to become segmental. Importantly, however, none of the patients developed generalized dystonia regardless age of onset and despite the long follow-up period.

Among the genetic primary dystonias, craniocervical dystonia has been mainly described in the context of DYT6 caused by *THAP1* (thanatos-associated protein 1) gene mutations. The mean age at onset in DYT6 is 24.4 years (range, 2-62 years),^4^ and the most common site of onset is the upper limb (47%) followed by cranial dystonia (25%), cervical dystonia (23%), and rarely leg dystonia (17%).^4^,^5^ In more than half of DYT6 patients, dystonia spreads to become generalized or multifocal, which contrasts our DYT24 patients.^2^,^4^–^8^ Recently, mutations in *GNAL* have also been identified to cause craniocervical dystonia.^9^ The average age of dystonia onset in those patients was 31.3 years (range, 7-54 years). Most carriers (82%) had onset in the neck, and most progressed to have dystonia at other sites. Cranial involvement was present in 57% of carriers, while brachial onset was not observed, and eventual arm involvement was seen in only 32% of carriers, in contrast to *THAP1* mutation carriers.^9^

Probably the most consistent feature in our patients with DYT24 was the presence of tremor. This cannot be screening bias, because an equal distribution of patients with and without tremor was screened. Interestingly, from 273 consecutive cervical dystonia patients, cervical dystonia with tremor was quite frequent (46%), and these patients more frequently reported a positive family history of dystonia or tremor (50%) than those without tremor (18%) in one study.^10^ Tremor as the predominant feature has been described only rarely in DYT6.^4^–^7^,^11^–^16^ Tremor has been described in DYT1, but the typical distribution, with dystonia starting in a lower limb and usually generalizing over some years, is quite distinct from the clinical picture in these *ANO3* patients.^17^ Also, in the patients with *GNAL* mutations, tremors have not been reported; however, further carriers are needed to reach a conclusion regarding the occurrence of tremor in these patients.^9^ Therefore, tremor may represent one of the major phenotypic differences between DYT6 and DYT24 craniocervical dystonia and a clue to test for *ANO3* mutations.

Two patients in our study, apart from tremor, had superimposed myoclonic jerks, and indeed myoclonus-dystonia (M-D) was considered as a possibility, although they tested negative for DYT11 (ε-sarcoglycan mutations). It is estimated that DYT11 accounts for about 30% of familial M-D cases, implying that further genes may cause an M-D phenotype.^18^ It is possible that *ANO3* mutations could account for at least some of those cases. DYT11 M-D presents with young-onset, action-induced, alcohol-responsive, brief, lightning myoclonic jerks that predominantly affect the cervical region (mainly retrocollic) and the upper limbs and less frequently affect the legs, while dystonic posturing may be present.^18^–^23^ In our patients, no such brief, shock-like myoclonic jerks were noted clinically or in electrophysiology. Thus, a case could be made for testing DYT11-negative patients with M-D for *ANO3* mutations.

Some of the patients with *ANO3* mutations were affected by tremor as the sole initial manifestation without or (later) with very mild dystonic posturing that was misdiagnosed as ET. These patients are important: first, they highlight the caveats in the current classification of dystonic tremor that requires the presence of clear dystonic posturing for diagnosis, which, if mild, can be missed on examination and may lead to misdiagnosis.^24^ Second, patients with genetically defined dystonia and prominent tremor are excellent candidates for electrophysiological studies to define laboratory criteria for the diagnosis of this tremor. Moreover, because patients with primary young-onset or adult-onset dystonia might present with an asymmetric arm and head and voice tremor without clear dystonic posturing, they could be misdiagnosed with ET, as in Family 3. The observations in our patients support the suggestion that patients who have isolated head tremor and head or voice tremor before the appearance of (or more severe than) hand tremor should be cautiously examined for even mild signs of dystonia (such as only mild dystonic finger posturing) before they are diagnosed with ET.^8^

The identification of the *ANO3* gene needs to be confirmed from independent studies, which also would allow an estimation of its frequency compared with other dystonia genes. Moreover, additional families are needed to unravel the full phenotypic spectrum of DYT24. Although the pathophysiology is not clear, the function of this gene is fascinating, because, for the first time, it implicates ion-channel dysfunction as a pathophysiological mechanism in dystonia.

## Legends to the Videos

**Video 1**. Family 1: Segment 1A shows patient II-4 at the age of 69 years (his symptoms having worsened from the earlier report at age 55 years)^3^, with torticollis to the left and tremor, blepharospasm, oromandibular movements, laryngeal dystonia, and a mild tremor on the left arm only with action. Segment 1B shows patient III-7 at the age of 36 years with tremulous torticollis to the right, laryngeal dystonia, and no arm tremor but a mild dystonic posturing of the arms in certain positions (the patient at age 24 years had milder symptoms and no laryngeal involvement).^3^

**Video 2**. Family 2: Segment 2A shows patient IV-1 with oromandibular movements, laryngeal dysphonia, head tremor, torticollis to the right, torticollic and retrocollic myoclonic jerks, and finger and arm myoclonus more on the left. Segment 2B shows patient IV-2 with mild dystonic finger posturing and mild postural tremor on the right hand.

**Video 3**. Family 3: Segment 3A shows patient II-1 with head tremor, mild torticollis to the right, and laryngeal dystonia. Segment 3B shows patient III-1 with arm tremor more on the left, which increases on action, and occasional myoclonic jerks on both arms.
